# Chicago Gynæcological Society; Meeting November 19th

**Published:** 1887-01

**Authors:** W. W. Jaggard

**Affiliations:** 2330 Indiana Avenue


### TRANSACTIONS OF THE CHICAGO GYNÆCOLOGICAL SOCIETY.

*Regular Meeting, Friday, November 19th, 1886.*—THE PRESIDENT, CHARLES WARRINGTON EARLE, M.D., in the chair.

DR. W. W. JAGGARD read a paper, entitled:

### A CASE OF CHRONIC INVERSION OF THE UTERUS, OF TWENTY-ONE MONTHS' STANDING, REDUCED BY COLPEURYSIS.—ABSTRACT.

*History.*—E. S., 36 years old, German; married at the age of 22 years; seven children, no miscarriages. Her first six confinements were normal. She was in the habit, common among German peasant-women, of rising upon the third day and of making up her own bed. In each of her labours she was attended by a midwife.

Her seventh confinement occurred in October, 1884. According to the statement of the patient and the attendant midwife the delivery of the child was normal. The placenta was removed, as in the six former labours, by traction on the cord. During the labour and the *puerperium* no unusual loss of blood was observed, and the patient does not remember any extraordinary sensations of pain or faintness. The midwife consulted a physician on the second day of the lying-in period, with reference to the sudden development of high bodily temperature. On the same day a well-known obstetrician saw the case. He made the diagnosis of puerperal fever, instituted the usual plan of treatment, but declined further connection with the case as he feared the infection of his regular puerperal patients, of whom he had a large number. No examination of the uterus, either by abdominal palpation or vaginal exploration, was made. On the third day an equally competent practitioner inspected the patient, confirmed the diagnosis of puerperal fever, and gave directions with reference to treatment. The contour of the uterus was not investigated either through the abdominal parietes or by the vagina. He continued to visit the patient for eight days, when he pronounced her convalescent. At the expiration of three weeks the woman rose from her bed for the first time, when she observed a fleshy tumor protruding from the vulva. Seven weeks after delivery she resumed her work as a washer-woman. She suckled her child fourteen months. During this period painful coitus and the sensation of the presence of a foreign body within the vagina were the only symptoms which attracted her attention to her condition. She noticed no fluor, no hemorrhage, and felt no pain except during coitus. The sexual act was not attended by any perceptible loss of blood. On account of the two symptoms mentioned she sought medical advice. The fleshy mass, situated entirely within the vagina, was supported by a large sponge.

The child was weaned in December, 1885. About March 17th, 1886, she experienced severe metrostaxis, entirely without pain, and lasting six days. She supposed menstruation had been re-established, and gave the subject no further thought. About April 15th another severe hemorrhage occurred, painless, and lasting one week. On May 28th she came under the writer's observation, and was admitted into the wards of Mercy Hospital. She sought relief, as she very distinctly expressed it, on account of painful coitus, the sensation of the presence of a foreign body within the vagina, and the excessive loss of blood during her last two menstrual periods. The woman was of medium size and height, with well-developed muscles and clavicles like a man's. She presented evidence of marked anæmia.

*Diagnosis.*—Bi-manual palpation revealed a pyriform tumor, the size of a hen's egg, protruding through the *os uteri.* The base of the tumor rested upon the pelvic floor, and upon coughing or straining appeared at the genital fissure. A shallow sulcus between the pedicle of the tumor and the walls of the cervical canal extending around the left semi-circumference of the canal, could be felt by the finger and traced with the sound. On the right side, no sulcus could be detected, and the membrane covering the tumor was reflected directly upon the external os. The long axis of the tumor was deflected to the left of the median line. The *corpus uteri* was absent from the normal position. The tumor, insensitive to pressure, was covered with a soft, villous membrane, and possessed the consistence of an œdematous myoma. The enveloping membrane was of a bluishred color, presenting some spots of superficial ulceration, and bled upon the slightest touch. Tubal ostia were nowhere visible. Traction of the tumor downwards caused the sulcus on the left side to disappear entirely, an important diagnostic sign of inversion of the uterus, to which Carl Braun, Robert Barnes and Schroeder in particular have called attention. Reamy, of Cincinnati, has recently described a sign which might have furnished corroborative evidence at this stage of the diagnosis in the case under consideration. Reamy says that when the tumor, grasped by the fingers within the vagina, can be easily rotated on its vertical axis, it is probably a polyp, since such rotation could not occur to any marked extent in an inverted uterus, stiffened as it is by its muscular walls and the thick, strong, fibrous guy-ropes furnished by the broad ligaments.

To make the differential diagnosis between inversion of the uterus and a pedunculated fibroid, positive, the patient was etherized. A sound in the bladder and a finger in the rectum were easily approximated above the tumor. The funnel-shaped cavity at the seat of inversion was easily recognized by the finger in the rectum, and by the hand on the abdomen in bimanual palpation.

No appearances were present that would indicate the invasion of the uterine walls by any new formation.

*Treatment.*—The patient was etherized, the contents of the rectum and bladder were evacuated, and the genitalia disinfected. The right hand was passed into the vagina, "and with the fingers and thumb encircling the portion of the body close to the seat of the inversion, the fundus was allowed to rest in the palm of the hand. This portion of the body was firmly grasped and pushed upward, and the fingers were then immediately separated to their utmost; at the same time the other hand was employed over the abdomen in the attempt to roll out the parts forming the ring, by sliding the abdominal parietes over its edge." At the expiration of forty-five minutes, the writer's right hand was almost powerless, and Dr. E. C. Dudley kindly relieved him. Dr. Dudley gave up the attempt at reduction after thirty minutes' trial, fearing perforation of the fundus. Apparently not the slightest progress in the reinversion of the organ had been made. Some hemorrhage occurred as the result of manipulation, although the fundus had been enveloped with absorbent cotton and gauze. The manœuvre was repeated on the following day, under the same conditions, through the same period of time, with no more favorable result.

Emmet's method was then abandoned for the following reasons: The separation of the fingers to their utmost had no effect whatever in the dilatation of the *os externum*. As pointed out by Fenger, and as brief reflection will convince the most casual observer, mere extension of the fingers can have but little effect in the dilatation of the cervix, owing to the relatively feeble character of the extensor muscles of the fore-arm. The necessary manipulation of the congested *mucosa*, even when protected by cotton or gauze, caused a loss of blood of moment in an already anæmic woman. The uterine musculature had evidently undergone fatty degeneration and there was serious danger of perforation. Finally, there was reason to entertain fear as to the patient's power to endure the shock from taxis and the effect of prolonged anæsthesia.

Compression of the body of the uterus opposite to each tubal ostium between the thumb and forefinger, so as to produce indentation of one side or the other—the Kimich Nœggerath method—was equally ineffectual.

On Sunday, May 30th, at the suggestion of Dr. W. H. Byford and Dr. Christian Fenger, the writer began an attempt to effect reinversion by colpeurysis. After the evacuation of the contents of the bladder and rectum, and disinfection of the genital canal, the colpeurynter was introduced while empty, so that it lay on the posterior wall of the vagina, and the *fundus uteri* was adjusted so that the long axis of the uterus and the axis of the pelvic inlet were coincident. The bag was then injected with water until it was fully distended. The patient was placed in bed in the dorsal decubitus. The instrument was removed at the expiration of twenty-four hours, and the genital canal disinfected. A *bacillus* containing thirty grains of iodoform was placed in the vaginal *cul de sac*, and the colpeurynter, after being cleansed, was reintroduced. Colpeurysis was continued in the manner indicated without interruption until June 9th. Very gradually the sulcus between the pedicle of the tumor and the neck of the uterus deepened, until on the eleventh day the organ was so far reinverted that the fundus was on the same plane with the *os externum*. During this period gentle efforts at taxis were made daily but without any apparent effect. No perceptible progress was made during the succeeding eight days. June 17th a serous fluid tinged with blood began to escape from the vagina, and it was thought the patient was about to menstruate. The colpeurynter was accordingly withdrawn. During the nights of June 18th and 21st the patient suffered severe uterine hemorrhages which threatened to prove immediately fatal. Hot vinegar was used as a vaginal douche, but did not prove so efficient a styptic as a hot saturated solution of alum. Menstruation ceased on

June 23d. On account of the hemorrhages, it was deemed inexpedient to expose the patient to the fatigue consequent upon any attempt to observe the *mucosa* during menstruation. During the subsequent nine days the writer was indisposed, so that the treatment by colpeurysis was resumed on July 2d. On examination, before replacing the bag, the inversion was found to be as complete and as irreducible as the day on which the treatment began. The uterus was gradually reinverted, as before, until on July 8th the fundus was on the same plane with the *os externum*. From the 8th until the 15th of July no apparent progress was made in reduction. On the evening of July 16th the writer was very much pleased to find the uterus completely reinverted, and the vaginal portion of the cervix occupying its normal position. The sound passed into the uterus to the extent of three and a half inches. The *corpus uteri* was felt on bimanual palpation, in a position of slight retroversion, below the promontory of the sacrum. The patient was not aware of any change in her condition. She said, however, that she had felt a sudden, sharp pain in the hypogastric region some four hours prior to the examination. Owing to the patient's enfeebled condition—due in the main part to anæmia—she was not permitted to leave her bed until July 18th.

The colpeurynter was in the vagina altogether thirty-three days. On three occasions during this period the bodily temperature rose to 102° F., but invariably fell to the normal after irrigation of the vagina and disinfection of the rubber bag. The presence of the colpeurynter in the vagina did not interfere at all with the functions of urination and defæcation. The writer desired to express in words his appreciation of the constant attention devoted to the somewhat tedious plan of treatment by Dr. Louis E. Lawson, late resident physician, Mercy hospital.

Dr. Alex. J. Stone, of St. Paul, kindly repaired the bilateral laceration of the cervix on July 20th. The operation was unusually difficult on account of the extent of the tear and the shortness of the vaginal portion. Dr. Stone's method of operative procedure differs materially from Emmet's, but its description is obviously out of place in the present report. The sutures were removed on August 4th, perfect union having been secured.

The patient, after leaving the hospital, gained rapidly in strength. Menstruation occurred September 26th; the process was painless, lasted four days, and the quantity of blood lost was normal. At the time of writing she has resumed her former occupation.

REMARKS.

The case is of particular interest with reference to: I, anatomy; II, symptoms, and III, treatment.

*I, Anatomy.*—The uterus was in a state intermediate between the second and third degrees of inversion. In the second degree of inversion—the incomplete inversion of Puzos, Levret, Leroux, Denncé, the third degree or perversion of Crosse—the anatomical limit of inversion has been indicated by Baudelocque as the vaginal insertion around the *cervix uteri.* Under these conditions, according to Veit and Freund, the cervical canal is intact, the uterus is only inverted as far as the internal os, and the uterine globe remains within the vagina. In the third degree, the complete inversion of Puzos, Levret, Leroux, and the utero-vaginal inversion of Denncé, the *corpus uteri* and *cervix uteri* are completely inverted, and the anatomical limit, as indicated by Levret, is the vaginal insertion at the vulvar orifice. Under these conditions the inverted uterus is also prolapsed, and protrudes beyond the plane of the genital fissure.

In the case under consideration the cervical canal was completely inverted on the right side, the cervico-uterine sulcus (Denncé) had disappeared, and the cervico-vaginal sulcus was shallow. On the left side the cervico-uterine and cervico-vaginal sulci were perfectly distinct. In consequence of the complete inversion of the right half of the cervix the long axis of the uterine globe was sensibly deflected to the left of the median line. The vaginal portion of the cervix was short, and lacerated on either side to the vaginal junction. The inverted uterus was perfectly mobile, and no trace of inflamatory infiltration could be detected about the pelvic peritoneum or in the connective tissue. The position of the ovaries, tubes and round ligaments could not be mapped out with any degree of certainty.

*II, Symptoms.*—The writer thought it was fair to assume that the inversion of the uterus, in the case under discussion, occurred at the time of delivery. The weight of probable evidence is in favor of this assumption. The inversion must have occurred before the third week following labour, because at that time the presence of an intra-vaginal tumor was discovered by the patient. This interval of three weeks was spent quietly in bed in the dorsal decubitus. The conditions for inversion would be at no time during this period so favorable as during or at the completion of the third stage of labour. During this period no cause adequate to the result was in operation. On the other hand, during or at the completion of the third stage of labour, all the causes and conditions known to be necessary to the production of inversion were present, *i.e.*, the enlarged and relaxed corpus, dilated cervix, traction on the cord, possibly, also fundal insertion of the placenta (Hennig) and paralysis of the placental site (Rokitansky). If this assumption be granted, the case demonstrates that inversion of the uterus may "take place without sufficient symptoms to attract attention or to indicate that anything has gone wrong." Dr. J. C. Reeve has already called attention to this subject, and has sustained the proposition just quoted by the citation of well authenticated cases in his classical essay, "Moot points in Regard to Inversion of the Uterus."

The patient a woman of at least average intelligence, and the midwife, a "qualified" practitioner, *i. e.*, examined and registered by the State Board of Health of Illinois, observed no symptoms sufficient to attract attention or to indicate that anything unusual had happened at the time of delivery. A well known and skillful obstetrician saw the case forty-eight hours after the probable time of the occurrence of the accident, and the absence of symptoms was so marked that the condition escaped his critical observation. Seventy-two hours after the probable time of occurrence of the accident the patient was seen by another thoroughly competent medical man, who also failed to recognize the complication upon his first, or upon any subsequent, visit.

Dr. Reeve's proposition has an important bearing upon the differential diagnosis between inversion of the uterus and sessile polypus, and indicates that no reliable evidence can be obtained from the history of the case.

*III, Treatment.*—Carl Braun, in 1851, introduced a simple, convenient and safe method of the vaginal tamponade (colpeurysis) by means of a caoutchouc bag (colpeurynter). The reduction of chronic inversion of the uterus by colpeurysis was inaugurated by a communication from Tyler Smith to the Royal Medical and Surgical Society of London, April 13th, 1858. In this communication Tyler Smith reports the reduction of a chronic inverted uterus by taxis in connection with continuous elastic pressure by means of Gariel's air-pessary.

Barrier, of Lyons, in 1852 employed an air-pessary to retain the uterus in position, but with no avowed intention of using continuous elastic pressure to effect reduction, as intimated by Dennce. M. P. Teale, Jr., of Leeds, and West, effected reductions of the inverted uterus in 1859 by Tyler Smith's method. It was reserved for Bockenthal, as remarked by Thomas, to demonstrate in the same year that reduction could be effected by the colpeurynter unaided by taxis.

As a matter of practical import, the colpeurynter used in the case described was a quadrilateral, caoutchouc bag, ten centimeters long, five centimeters wide when collapsed, and possessing a maximum circumference of twenty-one centimeters when distended. It is known in the shops as "No. 5 pear-shaped water-pessary." The selection of a properly shaped and properly sized instrument demands some care. Dr. Byford's treatise is the only American textbook on gynæcology which gives an adequate exposition of colpeurysis as one of the methods of reduction of chronic inversion of the uterus. This fact may be interpreted as indicating the method is not extensively practiced in the United States, and a survey of American medical literature upon this subject will serve to confirm such an opinion. In the very large majority of cases more heroic measures have been adopted. On the other hand, colpeurysis has largely replaced all other modes of treatment in Germany. Fritsch says, "Gradually almost all gynæcologists have gone over to Braun's colpeurynter." "The treatment with the colpeurynter is the sovereign method of treatment in cases of inversion of the uterus. Inversions yield to it which have resisted all other methods. The resistance which the cervix opposes may be so great that Muzeaux (four) forceps inserted into the portico tear out, and still the uterus remains unmoved. If colpeurysis is now resorted to, earlier or later a successful result is bound to follow, without the application of violence and without danger. It is therefore urgently advised to give up every attempt at forcible reposition of the uterus." He adds the significant sentence, "Colpeurysis cannot be held as without effect, even if the end is not immediately attained, it may be continued with interruptions fourteen days, yes, even three weeks." "The best method of treatment of chronic inversions," says Rheinstaeder, "is the introduction of a colpeurynter, which is gradually distended with water." Schroeder has repeatedly effected the reduction of the chronic inverted uterus after the failure of all efforts at manual reposition.

DISCUSSION.

DR. PHILIP ADOLPHUS: The author of this excellent paper has adopted in the reposition of the uterus of his patient, as efficient a mode of procedure as any hitherto in use. It is also the safest mode of replacing the organ. In the treatment of chronic inversions, success has followed all methods of replacement, whether effected gradually or rapidly. But forcible taxis ought to be the last resource, when gentler and as efficient means are exhausted It may lead to laceration of the vagina, peritonitis and death. Gradual pressure, sustained or interrupted, solid or elastic to which taxis has been added, has been equally successful, and has been practiced since 1858. It is absolutely safe. In some cases air pessaries or other elastic contrivances have been left in the vagina constantly, or have been replaced at intervals, for a period of three to eighteen days, and *uteri* have been returned by this method, which were inverted from one to fifteen years. The essential to success in the return of an inverted uterus is patient, gently continued manipulation of *some portion of the uterus*, by the fingers in the vagina with the application of the other hand externally to overcome the constriction of the cervix, and to prevent the forcible elongation of the vagina. A small hand, which observes the course of the pelvic axis, and avoids the promontory of the sacrum, and goes on one side of it, is also an element of success. Old adhesions opposing reduction of the inverted uterus are rarely present. An inflammation of the serous tissue in some portion of the pelvis may, however, be present as a complication, for this is an extremely common affection in all kinds of pelvic disease. Doubtless, in cases in which peritonitis followed manipulations, a chronic or subacute inflammation of the serous tissues was the predisposing cause. However, the most interesting portion of this subject to me is that of diagnosis, *in all tumors lying in the vagina*, which do not pathologically implicate that organ and the vulva. A correct diagnosis in inversion of the uterus is absolutely essential to treatment, and the safety of the patient The question of differential diagnosis between inversion of the uterus and polypi and fibroids is almost daily presented to the gynæcologist for solution. Not much reliance can be placed on the history in *chronic* inversion, for the diseases present similar symptoms. The size of an inverted uterus of some standing is scarcely larger, and is often smaller than in the natural state. It is desirable to look on the case under examination as one of inversion as long as any doubt exists. The bowels and the bladder should be emptied and the patient examined under ether. It is certainly *not* a case of inversion, when by bimanual *palpation*, with fingers in the vagina, fingers or hand into the rectum, or sound in the bladder, the *unimpaired roundness* of the uterus presents itself for palpation, either in the normal or retroverted position. In the just mentioned condition, if the sound enters the uterus two and a half inches or more, the uterus merely contains a fibroid or polypus which emerges from the cervix. The diagnosis may be rendered more difficult if no opening in the *cervix uteri* can be found, the cavity having been agglutinated by previous inflammation to the polypus. Here downward traction of the vaginal tumor to the vulva, by a vulsellum, as recommended by Susdorff, and I copy his words, will at once confirm the presence of a polypus. "For the relations of the parts to each other, as they existed in the vagina, will be greatly changed when exposed to view. The lips of the cervix which surrounded the pedicle will have disappeared, having also become inverted, and along with it, probably, the vagina at its junction with the neck." The insinuation of the sound into the uterus will at once confirm the information procured by bimanual palpation. If the same manner of examination discloses the body of the uterus indented or cupped, we have a partial inversion, either with or without a fibroid, a condition which is not as infrequent as is generally supposed. The presence of a tumor in the vagina, the absence of the *fundus uteri* in the abdomen, and the presence in its place of a well defined ring or cup-shaped cavity, unmistakably announces an inversion of the uterus; traction confirms the diagnosis. An incision, not a puncture, along the sides of the tumor, after the patient emerges from the ether, will at once show whether we have to deal with the fundus of the organ or a polypus.

In the one case it will induce pain, in the other it will prove painless. In the former it will relieve the congestion and possibly lead at once to its reposition, or prepare for its successful replacement in the future.

DR. H. P. MERRIMAN: I would like to ask whether, after the uterus had been partially restored so that the fundus was on a level with the lips, and the colpeurynter seemed to do no good for eight days following, taxis would not probably have promptly, almost immediately, accomplished the remaining portion of the work?

DR. H. T. BYFORD: Every method has danger, and there was one danger in this method which should be mentioned, that is the danger of sepsis or resorption of decomposing secretions. That there was danger even in this admirably managed case was evidenced by the rise in temperature, followed by the disappearance or decline in temperature on cleansing the bag and vagina. I have seen the immediate decline of fever by washing out the uterus when enlarged and filled with decomposing matter. I object to the introduction of the hand into the rectum to diagnose a case of inversion, as suggested by Dr. Adolphus. I consider it a dangerous practice because it does a violence to the part which sometimes has done an irreparable injury, and is unnecessary.

DR. W. H. BYFORD: With reference to the subject of inversion, and more particularly the diagnosis, there are two points which I think are very important, in addition to those mentioned by Dr. Jaggard. In cases of polypus attached to the neck of the uterus and filling up a good part of the vagina, I think the uterus is always enlarged and may be palpated above the pubes. Another point in the diagnosis is the difference in the sensation imparted to the examining finger. A polypus feels as if covered by a shining, smooth membrane, unless it is decomposed, while the surface of the uterus gives the sensation of pushing the finger into plush or velvet. I give these two points of diagnosis as the results of my own observation, and as being usually present. With reference to the mode of reducing inversion, I will give some of my own experiences during the last thirty years. In the year of 1859-60, I had a patient sent to me from Lafayette, Ind., with a chronic inversion of the uterus, which I attempted to reduce. I had just read a long treatise on the subject by Dr. White, of Buffalo, and Drs. Thomas and Emmet were then beginning to talk and write about these things, and I went at it with considerable enthusiasm. I got up the cup that Dr. Jaggard mentioned, and I also got a large rectal bougie, an instrument which Dr. White had praised very highly in his first operations, and I made the first attempt, lasting about an hour and three-quarters, and when I got through I was worse off than the patient, although she was pretty badly used up. I waited two or three weeks and made another attempt, but after a protracted effort I found my finger passing through the *fundus uteri.* I had been as cautious about the force as I could be, making the effort as gradually as possible, but I perforated the fundus. I fully expected that the damage done would be fatal to the patient, but it did not produce any bad effects whatever, and she entirely recovered in two weeks and went home. Two years later she came to see me again, but did not wish to have another effort made to have the uterus reduced. Two years later the uterus was found in its normal position. I saw the patient and her physician, and I am certain that nothing had been done to reduce it. I tried two other cases, and made the same efforts, but without success. I then concluded that it was hardly worth while to make trials of that nature again, and in the next case I tried the colpeurysis treatment. For some days I was nonplused, from want of experience, as to the mode of placing the instrument in the vagina. I used the quadrilateral colpeurynter, and after I placed it in the vagina I found the next day that I had gotten it under the uterus lengthwise, that the fundus was directed toward the vulva, and the neck directly backwards; I was merely compressing the body of the uterus against the *symphysis pubis.* I reflected considerably before I could get the right idea as to the manner of placing the instrument in the vagina. Finally I pushed back the fundus until the axis of the uterus corresponded to the axis of the superior strait, and then introduced the colpeurynter as has been described by Dr. Jaggard, and applied the force. The next day when I came back I found there had been some impression produced, and I went on with the use of it, taking it out every day and replacing it in this manner until in seven and a half days the inversion was reduced. The patient was a poor woman, and it was necessary for her to take care of her child. She did so, attended to it in every way and also cooked three meals a day for her husband. She was on her feet nearly the whole day time, and yet the instrument acted as well as if she had been lying in bed. Three out of the five cases I have operated on have been as painless as this. I should judge that a young primipara would probably suffer more from the use of the colpeurynter than one who had had children. I have now reduced five cases of inversion by the colpeurynter, and have not failed in any case since I commenced using the instrument. The first case of inversion I had I amputated the uterus. And in considering the matter since I doubt if any other treatment could have been adopted which would have been effectual. The uterus and vagina were both inverted, the whole vaginal canal was entirely outside of the body and the uterus hung down from it, both making a tumor nine inches long. The uterus was very much enlarged in consequence of its being dependent for so long a time. I was in consultation with two German physicians of this city, and they suggested, as the patient was living a miserable life and would die before long, we should cut it off. After half an hour's use of the ecraseur it was removed. We amputated a little below the center of the cervix. There was no bleeding, nothing to give rise to uneasiness. We pushed the vagina back again, put the parts in place, and the patient recovered in the course of a month. Having spoken of one spontaneous cure, I will tell you of a patient that I attended in Mercy Hospital in 1864-5 whose uterus was much in the same way as the one that I first operated on, coming out entirely beyond the vulva and dragging down the vagina very low, so that there was simply a circular sulcus between the labia and the vaginal wall. I tried to restore it by manipulation, and failed; I proposed to amputate it, but the patient would not consent. Meantime one of the Internes had fallen in love with her, and they went off to Missouri and got married. About six years afterwards the doctor came back, and told me that he had a son and his name was Byford. Upon inquiry I found that the child had been borne by this woman. One case of inversion occurred in my own practice. I attended the patient during confinement, and so far as I know she had no difficulties whatever for seven or eight days. By that time I was on my road to California, and I think Dr. Roler looked after her for some little time after I was gone. In two months I returned home and was informed that she had inversion of the uterus, which I did not believe. I went to see her, and found that she was suffering from complete inversion. That was one of the cases I cured by colpeurysis. When the inversion occurred I do not know. I am certain that I made two or three examinations, as I always did at that time, always one the second day after confinement. I did not notice anything of the kind, and yet it might have been commenced and finished afterwards. I saw a case with Dr. Henry T. Byford, which had been attended by a midwife, in which the inversion occurred so that the fundus could be touched through the mouth of the uterus, and it remained in that way two or three weeks. The patient was bleeding, but I believed the contraction of the mouth of the uterus was sufficient to prevent it coming through. I advised ergot, and in a few days the uterus was in its proper position.

DR. EDWARD WARREN SAWYER: One point is the persistence that one can observe in applying the colpeurysis, without a fatal result following. The interesting case that Dr. Byford has spoken of last also shows the possibility of the obstetrician seeing nothing in the first few days of the puerperal state to suggest that anything has gone wrong. Cases are recorded in which the inversion has taken place without the obstetrician knowing it. In the fatal case that occurred in my practice the symptoms were so profound that it was impossible to overlook it, and I think the diagnosis of recent puerperal inversion of the uterus is much easier than of chronic inversion. In the case which occurred in my practice, the rim of the crater marking the upper border of the uterus, which I palpated through the abdomen, was fully as large as a common bowl, and its edges were very sharply defined. In addition to that the fundus could be distinctly felt through the os uteri. One feature of the paper which is by no means the least to be commended is the very admirable and graphic way in which the case was presented.

DR. H. P. NEWMAN: My experience has been limited, but I remember a single case, in which I assisted a surgeon of this city in attempting the reduction of a chronic inversion of the uterus. It was in a hospital, where they had every facility for the operation and it could be proceeded with leisurely. Some two hours were taken up with the various devices for reducing the inverted fundus, all of which were of no avail. There was a complete inversion of the uterus but not of the vagina, and I think previous to the attempt at reduction a fibroid was removed from the fundus of the uterus. No further myomatous condition was discovered at the time, but the difficulty was exceedingly great in this case and nothing whatever was accomplished. I have no knowledge of the subsequent condition of the patient, whether she suffered materially from this, or whether she was afterwards successfully operated on, or the uterus amputated.

THE PRESIDENT asked Dr. Jaggard what means he used to disinfect the colpeurynter.

DR. JAGGARD replied that he washed it thoroughly with soap and warm water, afterwards disinfecting it with a five per centum solution of carbolic acid. The vagina was irrigated with a two per centum solution of carbolic acid, and a bacillus of iodoform introduced.

DR. ADOLPHUS, in reply to Dr. Henry T. Byford: In complicated cases of tumor in the abdomen or pelvis I would not do without the introduction of the hand into the rectum. I am not alluding to Simon's method, putting the hand in as far as the elbow, but I am talking of the hand. And when the patient is under ether, it can be done easily. It depends upon the size of the hand, perhaps, but with a hand well greased and introduced slowly it does a great deal of good and gives an immense deal of information which we cannot get in any other way. I examine every case, without exception, *per rectum*, with the finger.

THE PRESIDENT asked Dr. W. H. Byford if he regarded it good practice, after all ordinary means had been exhausted and the uterus was still inverted, to amputate?

DR. W. H. BYFORD: When all other measures have failed to effect the object and the patient is suffering so much as to make relief imperative, yes.

THE PRESIDENT: I saw Prof. Chiari, in Florence, operate upon a case of that sort. He placed a silver wire around the uterus and left it in position and the parts gradually sloughed away.

DR. W. H. BYFORD thought that mode of operating upon the uterus bad, that it would have been better to have used the wire ecraseur to stop circulation, and cut it off. But a sloughing mass in contact with the parts would be likely to produce pyaemia.

W. W. JAGGARD, M.D.

2330 Indiana Avenue.

